# Chalcogen Bonding due to the Exo-Substitution of Icosahedral Dicarbaborane

**DOI:** 10.3390/molecules24142657

**Published:** 2019-07-23

**Authors:** Jindřich Fanfrlík, Drahomír Hnyk, Pavel Hobza

**Affiliations:** 1 Institute of Organic Chemistry and Biochemistry of the Czech Academy of Sciences, Flemingovo nam. 2, 166 10 Prague 6, Czech Republic; 2 Institute of Inorganic Chemistry of the Czech Academy of Sciences, 250 68 Husinec-Řež, Czech Republic

**Keywords:** sigma hole, heteroborane, co-crystal

## Abstract

Chalcogen atoms are a class of substituents capable of generating inner and outer derivatives of boron clusters. It is well known that chalcogenated boron clusters can form strong σ-hole interactions when a chalcogen atom is a part of an icosahedron. This paper studies σ-hole interactions of dicarbaboranes with two exopolyhedral chalcogen atoms bonded to carbon vertices. Specifically, a computational investigation has been carried out on the co-crystal of (1,2-C_2_B_10_H_10_)_2_Se_4_•toluene and a single crystal of (1,2-C_2_B_10_H_10_)_2_Te_4_.

## 1. Introduction

**Figure 1:**
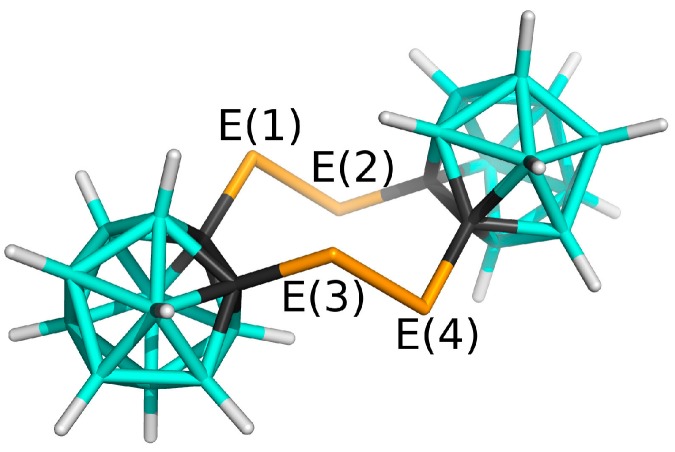
Molecular diagram of the **Se_4_C_4_** and **Te_4_C_4_** molecules that contain two icosahedral moieties connected by chalcogen bridges. The atom color coding is as follows: cyan—B; black—C; orange—E (E = Se, Te); white—H.

Polyhedral boron hydrides (boranes) are known for the presence of delocalized electron-deficient bonding. It is characterized by the aggregation of atoms to form 3-center-2-electron (3c-2e) bonds [1], which result in the formation of trigonal faces and hypercoordination. The three-dimensional deltahedral shapes typical of boron clusters are classified according to their formal electron counts, namely by the terms *closo*, *nido*, *arachno*, and *hypho* [2]. The *closo* clusters are of particular current interest as they exhibit especially high thermal and chemical stability. The number of vertices, n, can range from 5 to 12 with the formula B_n_H_n_^2−^, with the 12-vertex icosahedral cluster (an example in Figure 1), B_12_H_12_^2−^ (point-group symmetry *I_h_*), being the most common and most stable [3]. The replacement of one or more boron atoms at a vertex by atoms of other elements results in the formation of *closo* heteroboranes. For example, the replacement of two formally neutral BH groups by two CH^+^ moieties yields the neutral dicarbaboranes C_2_B_10_H_12_ in a variety of three isomers differing in the relative positions of the hypercarbon atoms within the icosahedral cage. The 1,2-isomer, the so-called o-carbaborane with *C_2v_* symmetry, is the least stable isomer and has the largest dipole moment [4] among them. This dipole moment directs from the middle point of the C-C vector toward the rest of the cluster. The fact that C vertices are the center of partial positive charge might be surprising because it contradicts the electronegativity concept. Such an electron distribution is, however, common in multicenter bonding [1]. Carboranes are considered 3D aromatic systems [5] and have rich substitution chemistry. Outer functionalization can be achieved by replacing terminal hydrogen atoms by various substituents to maintain the overall neutral charge. Chalcogen atoms belong to a class of substituents capable of generating derivatives of the icosahedral cage both as part of the cage [1] and as a peripheral group; the latter has so far been exemplified by thiolated carbaboranes. Very recently, a selenolated carbaborane with two SeH groups on both carbons has been prepared and structurally characterized [6]. However, the preparation of 1,2-(SeH)_2_-*closo*-1,2-C_2_B_10_H_10_ has been very difficult since the necessary intermediate for obtaining it, the lithium 1,2-diselenolato-1,2-dicarba-*closo*-dodecaboranate salt (Li^+^)_2_[1,2-(Se)_2_-*closo*-1,2-C_2_B_10_H_10_]^2−^, tends to dimerize in an oxidative manner [7] via two C-Se-Se-C diselenide bridges (see Figure 1). Analogous disulfide bridges are well known among cystein amino acids. It has been shown that the replacement of an interchain disulfide bridge with a diselenide bridge increases the lifetime of insulin without impairing its hormonal function [8]. The same dimerization as in (Li^+^)_2_[1,2–(Se)_2_-*closo*-1,2-C_2_B_10_H_10_]^2−^ also applies to (Li^+^)_2_[1,2–(Te)_2_-*closo*-1,2-C_2_B_10_H_10_]^2−^ [9]. Consequently, (1,2-C_2_B_10_H_10_)_2_E_4_ (E = Se, Te) have been obtained and structurally characterized as reported in references [7] and [9], respectively (see Figure 1). Note that such a molecular shape with the two o-carbaborane moieties and four chalcogens that form an eight-membered ring in a chair conformation also exist for E = S, but this structure has not been structurally described [10]. Cyclic molecules containing divalent chalcogen atoms have been receiving a great deal of attention as they can form columnar structures and even nanotubes via intermolecular diE-bonds [11,12,13].

Chalcogen atoms belong to a class of substituents capable of also generating icosahedral derivatives in which chalcogen atoms are part of the icosahedron, *closo*-1-EB_11_H_11_ (E = S, Se, Te) [2]. The substitution chemistry of *closo*-1-SB_11_H_11_ has also been researched, but to a lesser extent than in the case of *o*-carborane. Therefore, 12-ph-*closo*-1-SB_11_H_10_ was prepared and a very strong chalcogen bonding between a partially positively charged sulfur [14] atom and the π-electron cloud of the phenyl from the neighboring molecule in the crystal packing was detected [15]. Pnictogen bonding between 3D and 2D aromatics was also spotted in the cocrystal of *closo*-1,7-P_2_B_10_Cl_10_ and toluene [16]. Since toluene cocrystallizes with (1,2-C_2_B_10_H_10_)_2_Se_4_ (abbreviated as **Se_4_C_4_**) and (1,2-C_2_B_10_H_10_)_2_Te_4_ (abbreviated as **Te_4_C_4_**) crystallizes itself, we have carried out a computational investigation on these two crystals to examine the intensity of chalcogen (E-) and dichalcogen (diE-) bonding in the corresponding crystal packing. We have opted for these two crystals because the chalcogen atoms are located outside the icosahedron here in contrast to the previously studied heteroboranes with chalcogens directly involved in the icosahedron [15].

## 2. Methods

The molecular electrostatic potential (ESP) surfaces of isolated molecules were computed on the 0.001 a.u. molecular surfaces at the HF/def2-TZVP level using the Gaussian09 [17] and Molekel4.3 [18,19] programs.

The interactions in the crystal structures considered (CCDC codes 613,551 and 608,807) [7,9] were studied by employing a cluster model. The clusters were created around each unique molecule. The first layer consisted of molecules within 5 Å of the central molecule. Similarly, the second layer was formed by molecules within 5 Å of the first layer. Hydrogen atoms of the central molecule and the first layer were optimized by the DFT-D3/BLYP/DZVP method [20]. We selected the DFT-D3/BLYP/DZVP method because it makes it possible to obtain geometries close to more expensive calculations [20] for our extended clusters consisting of about 300–800 atoms. The resolution-of-identity (RI) approximation to the DFT method was also used. Due to the size of the second layer (comprising more than 1000 atoms), hydrogen atoms of the second layer were optimized by the semiempirical quantum mechanical PM6-D3H4X method [21]. Heavy atoms were kept in crystallographic positions. The clusters obtained were used for energy calculations. Interaction energy (ΔE) was computed as the energy difference between the energy of the dimer and the sum of monomer energies. The energies were determined using the DFT-D3/TPSS/TZVPP level. ΔE values were decomposed by symmetry-adapted perturbation-theory (SAPT) methodology. The simplest truncation of SAPT (SAPT0) decomposition [22] was performed with the recommended jun-cc-pVDZ basis set [23]. The noncovalent interaction index (NCI) [24,25] was calculated for selected motifs in order to elucidate all possible interactions in these motifs. It was carried out at the recommended B3LYP/6-31G* level [24]. The stability of the selected binding motifs was also examined by optimizations of all atoms. Here, we combined the DFT-D3/BLYP/DZVP method with the LBFGS algorithm and the strict optimization criteria (energy change < 0.0006 kcal mol^−1^, the largest gradient component < 0.12 kcal mol^−1^Å^−1^ and the root-mean-square gradient < 0.06 kcal mol^−1^Å^−1^). Vibrational frequencies were calculated numerically at the same level. Turbomole (7.0) [26], PSI4 [27], MOPAC2016 [28] and Cuby4 [29] programs were used.

## 3. Results and Discussion

### 3.1. Charge Distribution of Isolated Molecules

**Figure 2:**
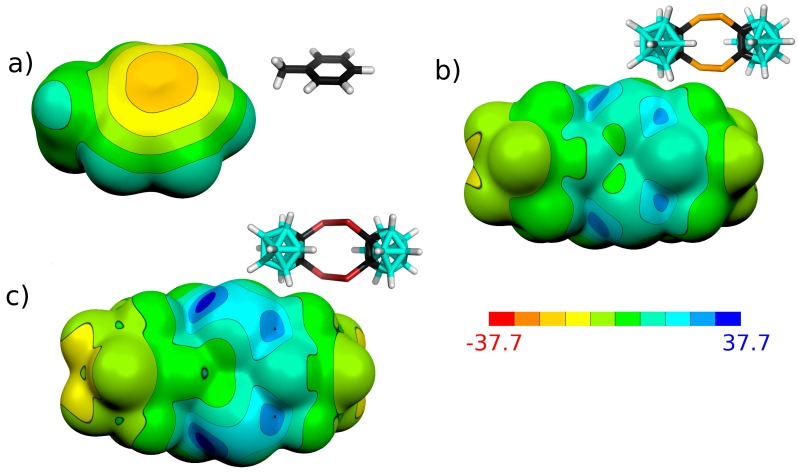
Molecular diagrams and computed electrostatic potential (ESP) molecular surfaces of toluene (**a**), **Se_4_C_4_** (**b**) and **Te_4_C_4_** (**c**). The ESP has been computed at the HF/def2TZVP level. The ESP color range is in kcal mol^−1^. The atom color coding is as follows: cyan—B; black—C; orange—Se; white—H; red—Te.

The charge distribution in isolated molecules has been studied by computing molecular electrostatic potential (ESP) surfaces (see Table 1 and Figure 2). The σ-holes [30] of the E atoms are the most positive areas of the molecular surfaces of the **Se_4_C_4_** and **Te_4_C_4_** molecules with large V_S,max_ values of 28.6 and 35.6 kcal mol^−1^. The **Se_4_C_4_** and **Te_4_C_4_** molecules are thus supposed to form strong σ-hole interactions. The higher value of **Te_4_C_4_** corresponds to the larger atomic number, smaller electronegativity, and larger polarizability of the Te atoms. The large V_S,max_ values of **Se_4_C_4_** and **Te_4_C_4_** molecules might be caused by the fact that the E atoms are bound to the C vertex of the carborane moiety. It has been shown that the S atom bound to the B vertex in 9,12-(SH)_2_-*closo*-1,2-C_2_B_10_H_10_ has a negative ESP molecular surface without a positive σ-hole in contrast to the carbon-bound S atom of 1,2-(SH)_2_-*closo*-1,2-C_2_B_10_H_10_, which has a σ-hole with the V_S,max_ value of 16.0 kcal mol^−1^ [31]. Analogically, halogenated dicarbaboranes could form halogen bonds only when they were bound to the C vertex [32]. It should be noted that the **Se_4_C_4_** and **Te_4_C_4_** molecules have zero dipole moments due to their symmetry, while the parental carborane molecule has a very large dipole moment. BH vertices have a negative ESP surface, as a result of which they could form attractive contacts with the σ-holes of the Se and Te atoms. The π ring of the toluene molecule has a more negative molecular ESP surface than the BH vertices (see Table 1 and Figure 2), and its interaction with the σ-holes should thus be more favorable.

### 3.2. Interactions in the Crystal of **Se_4_C_4_**•toluene and in the Single Crystal of **Te_4_C_4_**

**Figure 3:**
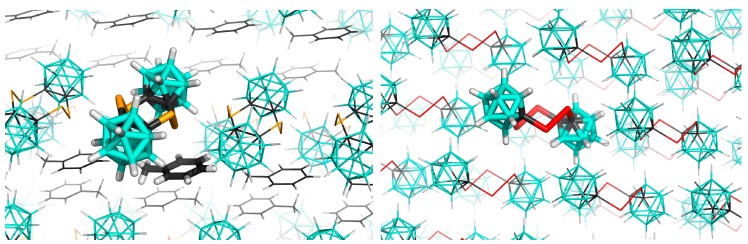
The crystal packing in the crystal of **Se_4_C_4_**•toluene (left) and the single crystal of **Te_4_C_4_** (right). The atom color coding is as follows: cyan—B; black—C; orange—Se; white—H; red—Te.

**Figure 4:**
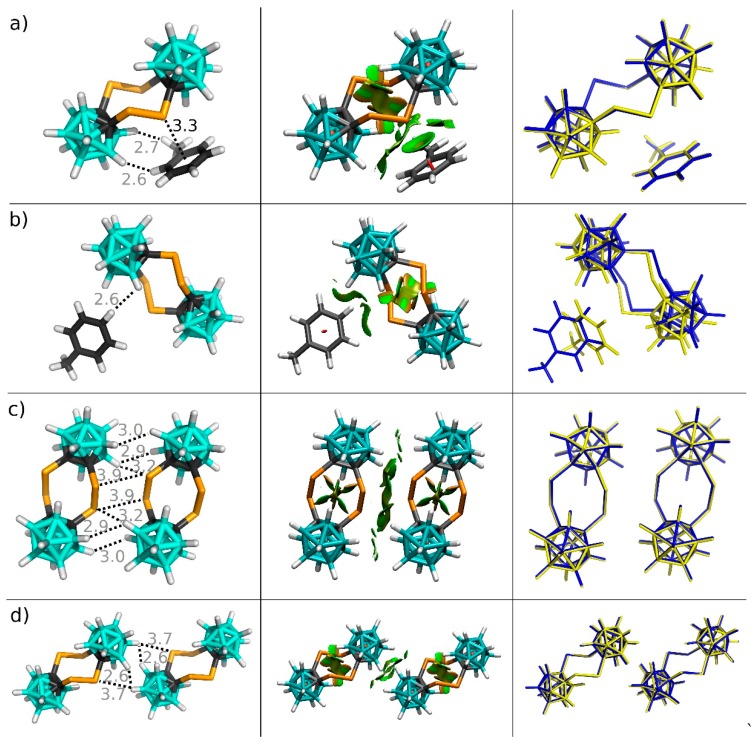
Four most stable binding motifs of the crystal structure of **Se_4_C_4_**•toluene (**a**), (**b**), (**c**) and (**d**). The left column: X-ray geometries (only the positions of H atoms optimized) and distances in Å. Distances larger than Σr_vdW_ are in gray. The middle column: NCI bonding isosurfaces. The NCI color scale is from −0.04 (blue) to 0.02 (red) a.u. The right column: A comparison of X-ray geometries (blue) with fully optimized structures in the gas phase (yellow).

The crystal packing of the studied crystals is shown in Figure 3. First, we focus on the crystal structure of **Se_4_C_4_**•toluene. The ΔE values of all crystal motifs are computed at the DFT-D3 level. Selected motifs are also examined by the SAPT0 method. The comparison of DFT-D3 and SAPT0 results verifies the accuracy of the selected DFT-D3 method (RMSE of 0.31 kcal mol^−1^). In the case of toluene, the sums of the ΔE values for the first and second layers are −28.50 and −1.90 kcal mol^−1^, respectively. The most negative ΔE value of the toluene molecules had the motif stabilized mainly by the C-Se···π E-bond with the separation between the Se atom and the center of the aromatic ring of the toluene being 3.30 Å, which corresponds to 91.7% of the sum of van der Waals radii (Σr_vdW_) [33] of the C and Se atoms (Figure 4A). This motif had a highly negative ΔE value, exceeding −9 kcal mol^−1^. It thus forms about 30% of the sum of ΔE values for the toluene molecule. The most important term of this motif in the SAPT decomposition is dispersion, followed by the electrostatic term. They form 64 and 27% of the attractive energy terms, respectively (Table 2). Similar results were reported for the EDA decomposition of the bifurcated type of Se···π interactions in diphenyl selenide [34]. The induction term which also includes charge transfer (orbital mixing) forms only about 9% of the attractive energy terms in the SATP decomposition, which agrees with findings that this contribution is smaller and does not determine the character of σ-hole interactions [35,36]. Additionally, this E-bond can be compared with the B-P···π pnictogen (Pn) bond found in the crystal of *closo*-1,7-P_2_B_10_Cl_10_•toluene, which had the ΔE value of −9.9 kcal mol^−1^ computed at the same DFT-D3 level, the length of 3.08 Å (88% of Σr_vdW_), and a larger contribution of the electrostatic term in the SAPT0 decomposition (35% of the attractive energy terms) [16]. It should also be mentioned that the free optimization followed by the frequency analysis confirmed that this motif represents true minimum on potential energy surface. The second most stable interaction of the toluene molecule of the studied crystal structure is a stacking motif, which has no contact shorter than Σr_vdW_ (Figure 4B) and the ΔE value of −4.07 kcal mol^−1^. In this motif, the dispersion is even more pronounced. It forms about 75% of the attractive energy terms in the SAPT decomposition. This motif was not stable in gas phase optimization and the comparison of the X-ray motif with the optimized geometry is shown in Figure 4. 

In the case of the **Se_4_C_4_** molecule, the sum of the ΔE values for the first and second layers are −85.96 and −3.61 kcal mol^−1^, respectively. Here, two motifs have highly negative ΔE values. One of them (i.e., the motif stabilized by the C-Se···π E-bond) was already described above. Interestingly, the **Se_4_C_4_** dimer has even more negative ΔE value exceeding −10 kcal mol^−1^, although it has no contact bellow Σr_vdW_ (see Figure 4c). It has two dichalcogen contacts of 3.87 Å (102% of Σr_vdW_) and two Se···H-B chalcogen contacts of 3.24 Å (108% of Σr_vdW_). Additionally, it has numerous B-H···H-B contacts with the H···H separation of about 3 Å (136% of Σr_vdW_). According to NCI analyses, these homopolar contacts also contribute to the binding. This is in agreement with the fact that the BH vector of dicarbaborane is another type of amphiphilic moiety [37], besides more the known CF_3_ moiety of trifluorotoulene [38]. Interestingly, the crystal structure also contain the **Se_4_C_4_** dimer motif stabilized exclusively via two homopolar B-H···H-B contacts (see Figure S2a) with the H···H separation of 2.60 Å (118% of Σr_vdW_). This contact has the ΔE value of −2.67 kcal mol^−1^ at the DFT-D3 level. Additionally, we have computed the ΔE value of a hypothetical dimer that has two B-H···H-B contacts (see Figure S2b) with the elongated H···H separation of 2.89 Å. This hypothetical dimer has the ΔE value of −2.22 kcal mol^−1^. We can thus speculate that the B-H···H-B contacts could form about 40% of the ΔE value for the interaction motif shown in Figure 4c.

**Figure 5:**
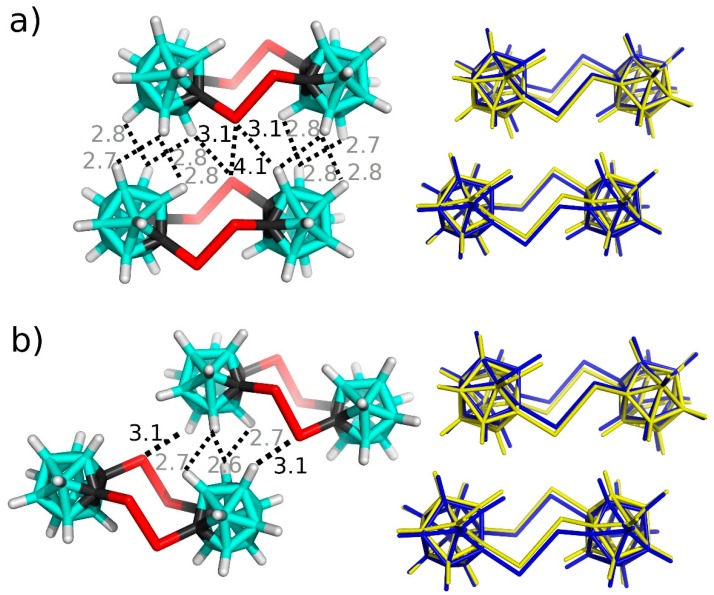
Two most stable binding motifs of the single crystal of **Te_4_C_4_** (**a**) and (**b**). The left column: X-ray geometries (only the positions of H atoms optimized) and distances in Å. Distances larger than Σr_vdW_ are in gray. The rigth column: Comparison of X-ray geometries (blue) with fully optimized structures in the gas phase (yellow). Distances are in Å. The positions of H atoms were optimized at the DFT-D3/BLYP/DZVP level.

In the second step, we focus on the single crystal of **Te_4_C_4_**. Here, the sums of the ΔE values of the first and second layers are −91.86 and −4.58 kcal mol^−1^, respectively. The most stable motif is stabilized by the C-Te···Te-C diE-bond with the Te···Te separation of 4.05 Å (98.3% of Σr_vdW_), two C-Te···H-B E-bonds with the Te···H separation of 3.13 Å (99.1% of Σr_vdW_), and numerous homopolar B-H···H-B contacts with the H···H separation of about 2.8 Å (127% of Σr_vdW_)—see Figure 5A. It has the ΔE value of −11.77 kcal mol^−1^, which is approximately 24.4% of the sum of the ΔE values of **Te_4_C_4_** considering that each molecule has this motif twice. The free optimization and frequency analysis have again confirmed the local minimum character of this motif. The second most stable motif has similar ΔE, specifically of −11.14 kcal mol^−1^. This motif has two C-Te···H-B E-bonds with the Te···H separation of 3.10 Å (98.1% of Σr_vdW_)—see Figure 5B. This motif also has several C-Te···H-B contacts with the Te···H separation of about 3.4 Å. Together, these diE- and E- bonds thus represent about 48% of the sum of the ΔE values of **Te_4_C_4_**. In contrast to unusual C-Te···H-B E-bonds, the Te···Te diE-bonds are considerably less rare; as an example, one can mention a very short Te···Te contact with the separation of 3.78 Å (91.7% of Σr_vdW_) in the crystal structure of 2,7-ditelluraocta-3,5-diyne [12].

In summary, we have shown that the dicarbaboranes with two exopolyhedral chalcogen atoms bonded to the carbon vertices have highly positive σ-holes and thus a great ability to form chalcogen bonds. A computational investigation carried out on the crystal of **Se_4_C_4_**•toluene and the single crystal of **Te_4_C_4_** has highlighted the importance of E···π, E···HB and E···E types of E- and diE-bonding.

## Figures and Tables

**Table 1:** The Dipole moment (μ) and the maximaum and minimum values of the electrostatic potential of the molecular surfaces (V_S,max_ and V_S,min_, respectively) computed at the HF/def2-TZVP level. Energies are in kcal mol^−1^ and μ in D.

	μ	V_S,max_	V_S,min_
Toluene	0.4	14.6	−19.8
**Se_4_C_4_**	0.0	28.6	−8.2
**Te_4_C_4_**	0.0	35.6	−10.1

**Table 2:** Computed DFT-D3/TPSS/TZVPP interaction energy (ΔE) values for the most stable motifs in the crystal of **Se_4_C_4_**•toluene. Interaction energies were decomposed into electrostatic (E_elec_), induction (E_ind_), dispersion (E_disp_), and exchange (E_exch_) contributions using the SAPT0/jun-cc-pVDZ methodology. Energies are in kcal mol^−1^. The relative values in parentheses show the contribution to the sum of all the attractive energy terms of SAPT0.

Interaction Motif	ΔE					
	DFT-D3	SAPT0				
		Total	E_elec_	E_ind_	E_disp_	E_exch_
**Se_4_C_4_···toluene** (E-bonding) ^1^	−9.22	−9.28	−5.47 (27%)	−1.81 (9%)	−12.66 (64%)	10.66
**Se_4_C_4_**···**toluene** (stacking) ^2^	−4.07	−3.62	−1.32 (19%0	−0.39 (5%)	−5.13 (75%)	3.22
**Se_4_C_4_**···**Se_4_C_4_** (stacking_1) ^3^	−10.23	−10.27	−5.06 (22%)	−1.53 (7%)	−16.04 (71%)	12.36
**Se_4_C_4_**···**Se_4_C_4_** (stacking_2) ^4^	−5.02	−5.43	−1.86 (19%)	−0.37 (4%)	−7.61 (77%)	4.41

^1^ The motif is shown in Figure 4a. ^2^ The motif is shown in Figure 4b. ^3^ The motif is shown in Figure 4c. ^4^ The motif is shown in Figure 4d.

## Supplementary Materials

The following are available online, Figure S1: 2D-NCI plot of reduced density gradient (RDG) vs. sign(λ_2_)ρ, Figure S2: Binding motifs of **Se_4_C_4_** dimer stabilized via homopolar B-H···H-B contacts.
