# Supplementary material for: Chalcogen Bonding due to the Exo-Substitution of Icosahedral Dicarbaborane
Source: Molecules. 2019 Jul 23;24(14):2657. doi: 10.3390/molecules24142657 (PMC6680755; doi:10.3390/molecules24142657)
Supplement: Supplementary file 1 [file molecules-24-02657-s001.pdf]

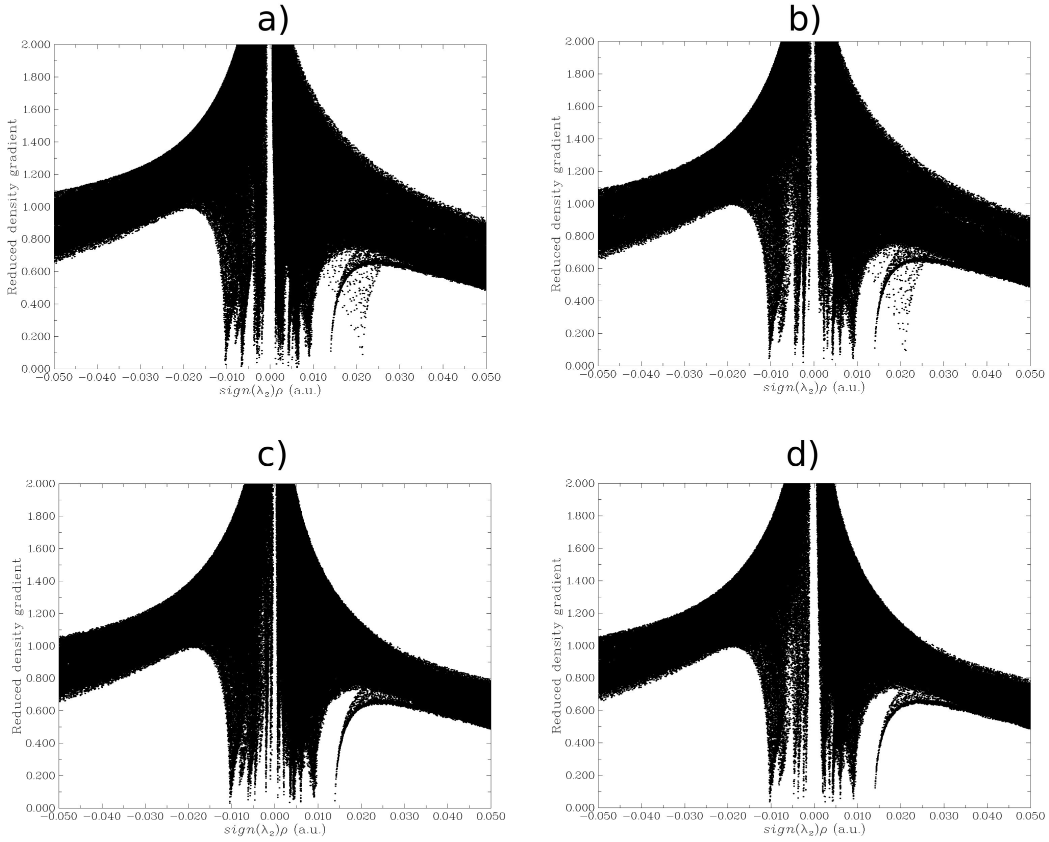

Figure S1. 2D-NCI plot of reduced density gradient (RDG) vs. sign(λ_2)ρ for motifs shown in Figure 4.

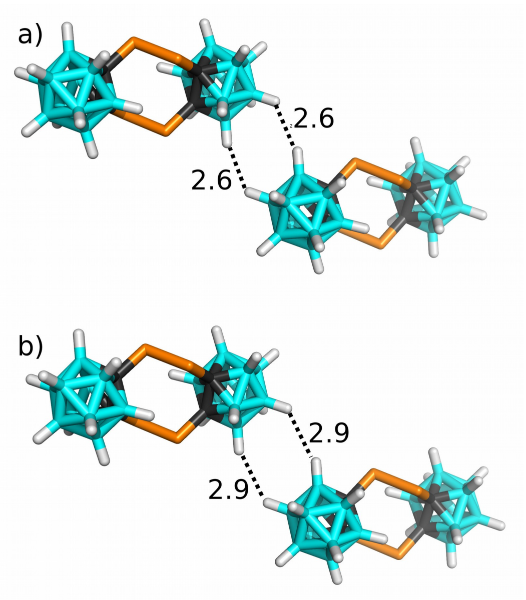

Figure S2. The X-ray (a) and hypothetical (b) binding motifs of **Se_4C_4** dimer stabilized via homopolar B-H···H-B contacts. The positions of H atoms optimized were optimized in the X-ray geometry. Distances are in Å. The atom color coding is as follows: cyan – B; black – C; orange – Se; white – H.

Se_4C_4···toluene E-bonding motif
Energy (DFT-D3/BLYP/DZVP): -6612317.1021883 kcal mol^{-1}
63

C	22.893000000	21.486000000	27.942000000
C	21.608000000	21.979000000	28.083000000
C	21.309000000	23.272000000	27.697000000
C	22.301000000	24.064000000	27.180000000
C	23.587000000	23.573000000	27.029000000
C	25.303000000	21.764000000	27.285000000
C	23.908000000	22.281000000	27.397000000
H	23.118000000	20.459000000	28.246000000
H	20.828000000	21.334000000	28.497000000
H	20.297000000	23.666000000	27.804000000
H	22.077000000	25.093000000	26.885000000
H	24.367000000	24.220000000	26.614000000
H	25.379000000	20.719000000	27.612000000
H	25.684000000	21.815000000	26.254000000
H	25.990000000	22.359000000	27.907000000
C	24.941000000	26.751000000	30.346000000
C	25.542000000	22.473000000	32.466000000
C	26.570000000	26.495000000	30.713000000
C	23.913000000	22.729000000	32.099000000
Se	23.505000000	25.976000000	31.398000000
Se	26.978000000	23.247000000	31.414000000
Se	23.341000000	23.823000000	30.598000000
Se	27.142000000	25.400000000	32.214000000
B	26.389000000	27.011000000	28.010000000
B	24.094000000	22.212000000	34.802000000
B	27.522000000	26.638000000	29.309000000
B	22.961000000	22.586000000	33.503000000
B	27.336000000	28.318000000	28.782000000
B	23.147000000	20.905000000	34.029000000
B	25.803000000	27.968000000	31.201000000
B	24.680000000	21.256000000	31.611000000
B	24.671000000	28.371000000	29.878000000
B	25.812000000	20.852000000	32.934000000
B	24.740000000	27.064000000	28.685000000
B	25.743000000	22.160000000	34.127000000
B	25.920000000	25.846000000	29.264000000
B	24.563000000	23.378000000	33.548000000
B	27.456000000	27.942000000	30.518000000
B	23.027000000	21.282000000	32.294000000
B	26.272000000	29.147000000	29.965000000
B	24.211000000	20.076000000	32.847000000
B	25.624000000	28.588000000	28.395000000
B	24.859000000	20.636000000	34.417000000
H	26.382000000	30.293000000	30.264000000
H	24.098000000	18.930000000	32.553000000
H	25.250000000	29.337000000	27.552000000
H	25.231000000	19.881000000	35.259000000

```
 H   26.581000000  26.627000000  26.902000000
 H   23.902000000  22.597000000  35.911000000
 H   28.477000000  25.940000000  29.243000000
 H   22.008000000  23.286000000  33.582000000
 H   28.229000000  28.874000000  28.225000000
 H   22.253000000  20.347000000  34.582000000
 H   25.565000000  28.115000000  32.354000000
 H   24.916000000  21.112000000  30.461000000
 H   23.628000000  28.855000000  30.175000000
 H   26.853000000  20.370000000  32.640000000
 H   28.367000000  28.125000000  31.258000000
 H   22.123000000  21.091000000  31.552000000
 H   23.749000000  26.665000000  28.177000000
 H   26.735000000  22.560000000  34.642000000
 H   25.756000000  24.684000000  29.171000000
 H   24.720000000  24.542000000  33.644000000
```

Se_4C_4···toluene stacking motif
Energy (DFT-D3/BLYP/DZVP): -6612312.03215757 kcal mol^{-1}
63

```
 C   22.893000000  21.486000000  27.942000000
 C   21.608000000  21.979000000  28.083000000
 C   21.309000000  23.272000000  27.697000000
 C   22.301000000  24.064000000  27.180000000
 C   23.587000000  23.573000000  27.029000000
 C   25.303000000  21.764000000  27.285000000
 C   23.908000000  22.281000000  27.397000000
 H   23.118000000  20.459000000  28.246000000
 H   20.828000000  21.334000000  28.497000000
 H   20.297000000  23.666000000  27.804000000
 H   22.077000000  25.093000000  26.885000000
 H   24.367000000  24.220000000  26.614000000
 H   25.379000000  20.719000000  27.612000000
 H   25.684000000  21.815000000  26.254000000
 H   25.990000000  22.359000000  27.907000000
 C   17.566000000  26.751000000  30.346000000
 C   18.167000000  22.473000000  32.466000000
 C   19.195000000  26.495000000  30.713000000
 C   16.538000000  22.729000000  32.099000000
Se   16.130000000  25.976000000  31.398000000
Se   19.603000000  23.247000000  31.414000000
Se   15.966000000  23.823000000  30.598000000
Se   19.767000000  25.400000000  32.214000000
 B   19.014000000  27.011000000  28.010000000
 B   16.719000000  22.212000000  34.802000000
 B   20.147000000  26.638000000  29.309000000
 B   15.586000000  22.586000000  33.503000000
 B   19.961000000  28.318000000  28.782000000
 B   15.772000000  20.905000000  34.029000000
 B   18.428000000  27.968000000  31.201000000
 B   17.305000000  21.256000000  31.611000000
```

B	17.296000000	28.371000000	29.878000000
B	18.437000000	20.852000000	32.934000000
B	17.365000000	27.064000000	28.685000000
B	18.368000000	22.160000000	34.127000000
B	18.545000000	25.846000000	29.264000000
B	17.188000000	23.378000000	33.548000000
B	20.081000000	27.942000000	30.518000000
B	15.652000000	21.282000000	32.294000000
B	18.897000000	29.147000000	29.965000000
B	16.836000000	20.076000000	32.847000000
B	18.249000000	28.588000000	28.395000000
B	17.484000000	20.636000000	34.417000000
H	19.009000000	30.293000000	30.263000000
H	16.724000000	18.929000000	32.553000000
H	17.877000000	29.339000000	27.551000000
H	17.857000000	19.882000000	35.258000000
H	19.206000000	26.627000000	26.902000000
H	16.528000000	22.597000000	35.911000000
H	21.099000000	25.942000000	29.233000000
H	14.629000000	23.286000000	33.573000000
H	20.860000000	28.869000000	28.233000000
H	14.881000000	20.345000000	34.584000000
H	18.191000000	28.115000000	32.354000000
H	17.545000000	21.113000000	30.461000000
H	16.255000000	28.858000000	30.176000000
H	19.480000000	20.372000000	32.641000000
H	20.991000000	28.129000000	31.256000000
H	14.745000000	21.097000000	31.552000000
H	16.376000000	26.664000000	28.170000000
H	19.361000000	22.558000000	34.640000000
H	18.383000000	24.684000000	29.170000000
H	17.347000000	24.541000000	33.644000000

Se_4C_4··· **Se_4C_4** stacking motif (first)
Energy (DFT-D3/BLYP/DZVP): -12883938.0424843 kcal mol^{-1}
96

C	10.191000000	26.751000000	30.346000000
C	10.792000000	22.473000000	32.466000000
C	11.820000000	26.495000000	30.713000000
C	9.163000000	22.729000000	32.099000000
Se	8.755000000	25.976000000	31.398000000
Se	12.228000000	23.247000000	31.414000000
Se	8.591000000	23.823000000	30.598000000
Se	12.392000000	25.400000000	32.214000000
B	11.639000000	27.011000000	28.010000000
B	9.344000000	22.212000000	34.802000000
B	12.772000000	26.638000000	29.309000000
B	8.211000000	22.586000000	33.503000000
B	12.586000000	28.318000000	28.782000000
B	8.397000000	20.905000000	34.029000000
B	11.053000000	27.968000000	31.201000000

B	9.930000000	21.256000000	31.611000000
B	9.921000000	28.371000000	29.878000000
B	11.062000000	20.852000000	32.934000000
B	9.990000000	27.064000000	28.685000000
B	10.993000000	22.160000000	34.127000000
B	11.170000000	25.846000000	29.264000000
B	9.813000000	23.378000000	33.548000000
B	12.706000000	27.942000000	30.518000000
B	8.277000000	21.282000000	32.294000000
B	11.522000000	29.147000000	29.965000000
B	9.461000000	20.076000000	32.847000000
B	10.873000000	28.588000000	28.395000000
B	10.109000000	20.636000000	34.417000000
H	11.635000000	30.292000000	30.260000000
H	9.348000000	18.931000000	32.552000000
H	10.506000000	29.334000000	27.549000000
H	10.476000000	19.890000000	35.263000000
H	11.831000000	26.631000000	26.901000000
H	9.152000000	22.592000000	35.911000000
H	13.726000000	25.944000000	29.232000000
H	7.257000000	23.280000000	33.581000000
H	13.480000000	28.867000000	28.225000000
H	7.503000000	20.356000000	34.586000000
H	10.818000000	28.117000000	32.350000000
H	10.165000000	21.107000000	30.462000000
H	8.879000000	28.856000000	30.167000000
H	12.104000000	20.368000000	32.645000000
H	13.612000000	28.134000000	31.257000000
H	7.371000000	21.090000000	31.555000000
H	8.999000000	26.668000000	28.175000000
H	11.984000000	22.556000000	34.637000000
H	11.010000000	24.684000000	29.167000000
H	9.973000000	24.540000000	33.646000000
C	2.816000000	26.751000000	30.346000000
C	3.417000000	22.473000000	32.466000000
C	4.445000000	26.495000000	30.713000000
C	1.788000000	22.729000000	32.099000000
Se	1.380000000	25.976000000	31.398000000
Se	4.853000000	23.247000000	31.414000000
Se	1.216000000	23.823000000	30.598000000
Se	5.017000000	25.400000000	32.214000000
B	4.264000000	27.011000000	28.010000000
B	1.969000000	22.212000000	34.802000000
B	5.397000000	26.638000000	29.309000000
B	0.836000000	22.586000000	33.503000000
B	5.211000000	28.318000000	28.782000000
B	1.022000000	20.905000000	34.029000000
B	3.678000000	27.968000000	31.201000000
B	2.555000000	21.256000000	31.611000000
B	2.546000000	28.371000000	29.878000000
B	3.687000000	20.852000000	32.934000000
B	2.615000000	27.064000000	28.685000000

B	3.618000000	22.160000000	34.127000000
B	3.795000000	25.846000000	29.264000000
B	2.438000000	23.378000000	33.548000000
B	5.331000000	27.942000000	30.518000000
B	0.902000000	21.282000000	32.294000000
B	4.147000000	29.147000000	29.965000000
B	2.086000000	20.076000000	32.847000000
B	3.499000000	28.588000000	28.395000000
B	2.734000000	20.636000000	34.417000000
H	4.256000000	30.291000000	30.268000000
H	1.972000000	18.929000000	32.556000000
H	3.128000000	29.341000000	27.552000000
H	3.100000000	19.896000000	35.268000000
H	4.456000000	26.632000000	26.901000000
H	1.778000000	22.597000000	35.912000000
H	6.350000000	25.943000000	29.232000000
H	-0.120000000	23.287000000	33.574000000
H	6.109000000	28.865000000	28.229000000
H	0.131000000	20.346000000	34.585000000
H	3.442000000	28.116000000	32.352000000
H	2.793000000	21.110000000	30.460000000
H	1.505000000	28.859000000	30.177000000
H	4.729000000	20.370000000	32.643000000
H	6.237000000	28.133000000	31.257000000
H	-0.008000000	21.097000000	31.553000000
H	1.624000000	26.663000000	28.169000000
H	4.611000000	22.557000000	34.634000000
H	3.637000000	24.682000000	29.167000000
H	2.600000000	24.540000000	33.645000000

Se_4C_4··· **Se_4C_4** stacking motif (second)
Energy (DFT-D3/BLYP/DZVP): -12883932.7807365 kcal mol^{-1}
96

C	10.191000000	26.751000000	30.346000000
C	10.792000000	22.473000000	32.466000000
C	11.820000000	26.495000000	30.713000000
C	9.163000000	22.729000000	32.099000000
Se	8.755000000	25.976000000	31.398000000
Se	12.228000000	23.247000000	31.414000000
Se	8.591000000	23.823000000	30.598000000
Se	12.392000000	25.400000000	32.214000000
B	11.639000000	27.011000000	28.010000000
B	9.344000000	22.212000000	34.802000000
B	12.772000000	26.638000000	29.309000000
B	8.211000000	22.586000000	33.503000000
B	12.586000000	28.318000000	28.782000000
B	8.397000000	20.905000000	34.029000000
B	11.053000000	27.968000000	31.201000000
B	9.930000000	21.256000000	31.611000000
B	9.921000000	28.371000000	29.878000000
B	11.062000000	20.852000000	32.934000000

B	9.990000000	27.064000000	28.685000000
B	10.993000000	22.160000000	34.127000000
B	11.170000000	25.846000000	29.264000000
B	9.813000000	23.378000000	33.548000000
B	12.706000000	27.942000000	30.518000000
B	8.277000000	21.282000000	32.294000000
B	11.522000000	29.147000000	29.965000000
B	9.461000000	20.076000000	32.847000000
B	10.873000000	28.588000000	28.395000000
B	10.109000000	20.636000000	34.417000000
H	11.635000000	30.292000000	30.260000000
H	9.348000000	18.931000000	32.552000000
H	10.506000000	29.334000000	27.549000000
H	10.476000000	19.890000000	35.263000000
H	11.831000000	26.631000000	26.901000000
H	9.152000000	22.592000000	35.911000000
H	13.726000000	25.944000000	29.232000000
H	7.257000000	23.280000000	33.581000000
H	13.480000000	28.867000000	28.225000000
H	7.503000000	20.356000000	34.586000000
H	10.818000000	28.117000000	32.350000000
H	10.165000000	21.107000000	30.462000000
H	8.879000000	28.856000000	30.167000000
H	12.104000000	20.368000000	32.645000000
H	13.612000000	28.134000000	31.257000000
H	7.371000000	21.090000000	31.555000000
H	8.999000000	26.668000000	28.175000000
H	11.984000000	22.556000000	34.637000000
H	11.010000000	24.684000000	29.167000000
H	9.973000000	24.540000000	33.646000000
C	11.427000000	16.296000000	30.346000000
C	12.027000000	12.019000000	32.466000000
C	13.055000000	16.041000000	30.713000000
C	10.399000000	12.274000000	32.099000000
Se	9.990000000	15.522000000	31.398000000
Se	13.464000000	12.793000000	31.414000000
Se	9.827000000	13.369000000	30.598000000
Se	13.627000000	14.946000000	32.214000000
B	12.874000000	16.557000000	28.010000000
B	10.580000000	11.758000000	34.802000000
B	14.007000000	16.184000000	29.309000000
B	9.446000000	12.131000000	33.503000000
B	13.822000000	17.864000000	28.782000000
B	9.632000000	10.451000000	34.029000000
B	12.288000000	17.514000000	31.201000000
B	11.165000000	10.802000000	31.611000000
B	11.156000000	17.917000000	29.878000000
B	12.297000000	10.398000000	32.934000000
B	11.225000000	16.610000000	28.685000000
B	12.228000000	11.706000000	34.127000000
B	12.406000000	15.392000000	29.264000000
B	11.048000000	12.924000000	33.548000000

B	13.941000000	17.488000000	30.518000000
B	9.512000000	10.827000000	32.294000000
B	12.757000000	18.693000000	29.965000000
B	10.697000000	9.622000000	32.847000000
B	12.109000000	18.133000000	28.395000000
B	11.345000000	10.182000000	34.417000000
H	12.868000000	19.838000000	30.262000000
H	10.586000000	8.476000000	32.549000000
H	11.743000000	18.882000000	27.549000000
H	11.717000000	9.430000000	35.261000000
H	13.066000000	16.175000000	26.899000000
H	10.386000000	12.140000000	35.910000000
H	14.964000000	15.485000000	29.238000000
H	8.495000000	12.829000000	33.577000000
H	14.713000000	18.418000000	28.223000000
H	8.738000000	9.896000000	34.584000000
H	12.051000000	17.664000000	32.350000000
H	11.402000000	10.655000000	30.458000000
H	10.115000000	18.401000000	30.170000000
H	13.338000000	9.912000000	32.634000000
H	14.847000000	17.677000000	31.259000000
H	8.604000000	10.641000000	31.553000000
H	10.232000000	16.212000000	28.172000000
H	13.218000000	12.108000000	34.640000000
H	12.248000000	14.228000000	29.166000000
H	11.209000000	14.087000000	33.643000000

Te_4C_4···Te_4C_4 dichalcogen bonding motif
Energy (DFT-D3/BLYP/DZVP): -869102.261560641 kcal mol^{-1}
96

C	40.865000000	26.548000000	27.742000000
C	38.214000000	30.576000000	30.054000000
C	40.086000000	27.470000000	26.548000000
C	38.992000000	29.654000000	31.248000000
B	41.772000000	25.632000000	25.276000000
B	37.306000000	31.492000000	32.520000000
B	40.849000000	24.305000000	26.095000000
B	38.229000000	32.820000000	31.701000000
B	40.079000000	25.295000000	24.814000000
B	38.999000000	31.830000000	32.982000000
B	41.734000000	27.050000000	26.365000000
B	37.344000000	30.075000000	31.431000000
B	41.927000000	25.406000000	27.020000000
B	37.151000000	31.718000000	30.776000000
B	40.303000000	24.923000000	27.646000000
B	38.776000000	32.201000000	30.150000000
B	39.186000000	26.274000000	27.395000000
B	39.892000000	30.851000000	30.401000000
B	39.139000000	24.858000000	26.326000000
B	39.939000000	32.266000000	31.470000000
B	39.038000000	26.495000000	25.613000000

B	40.041000000	30.629000000	32.183000000
B	40.617000000	26.961000000	24.977000000
B	38.461000000	30.164000000	32.818000000
Te	41.531000000	27.338000000	29.631000000
Te	37.547000000	29.786000000	28.164000000
Te	39.292000000	27.509000000	31.088000000
Te	39.786000000	29.615000000	26.708000000
H	42.699000000	25.448000000	24.558000000
H	36.379000000	31.676000000	33.238000000
H	41.142000000	23.163000000	25.956000000
H	37.936000000	33.962000000	31.840000000
H	39.782000000	24.862000000	23.750000000
H	39.296000000	32.263000000	34.046000000
H	42.557000000	27.899000000	26.487000000
H	36.521000000	29.226000000	31.309000000
H	42.910000000	25.126000000	27.620000000
H	36.168000000	31.998000000	30.176000000
H	40.235000000	24.328000000	28.666000000
H	38.844000000	32.796000000	29.130000000
H	38.377000000	26.631000000	28.177000000
H	40.701000000	30.493000000	29.619000000
H	38.202000000	24.122000000	26.337000000
H	40.876000000	33.002000000	31.459000000
H	38.052000000	27.002000000	25.203000000
H	41.027000000	30.122000000	32.594000000
H	40.708000000	27.796000000	24.141000000
H	38.370000000	29.329000000	33.655000000
C	34.009000000	26.548000000	27.742000000
C	31.358000000	30.576000000	30.054000000
C	33.231000000	27.470000000	26.548000000
C	32.137000000	29.654000000	31.248000000
B	34.917000000	25.632000000	25.276000000
B	30.451000000	31.492000000	32.520000000
B	33.994000000	24.305000000	26.095000000
B	31.373000000	32.820000000	31.701000000
B	33.224000000	25.295000000	24.814000000
B	32.144000000	31.830000000	32.982000000
B	34.878000000	27.050000000	26.365000000
B	30.489000000	30.075000000	31.431000000
B	35.072000000	25.406000000	27.020000000
B	30.296000000	31.718000000	30.776000000
B	33.447000000	24.923000000	27.646000000
B	31.920000000	32.201000000	30.150000000
B	32.330000000	26.274000000	27.395000000
B	33.037000000	30.851000000	30.401000000
B	32.283000000	24.858000000	26.326000000
B	33.084000000	32.266000000	31.470000000
B	32.182000000	26.495000000	25.613000000
B	33.185000000	30.629000000	32.183000000
B	33.762000000	26.961000000	24.977000000
B	31.606000000	30.164000000	32.818000000
Te	34.676000000	27.338000000	29.631000000

Te	30.692000000	29.786000000	28.164000000
Te	32.436000000	27.509000000	31.088000000
Te	32.931000000	29.615000000	26.708000000
H	35.848000000	25.453000000	24.562000000
H	29.525000000	31.670000000	33.246000000
H	34.287000000	23.163000000	25.959000000
H	31.083000000	33.967000000	31.845000000
H	32.925000000	24.866000000	23.747000000
H	32.444000000	32.263000000	34.048000000
H	35.701000000	27.900000000	26.489000000
H	29.668000000	29.223000000	31.302000000
H	36.056000000	25.137000000	27.625000000
H	29.308000000	31.993000000	30.174000000
H	33.373000000	24.336000000	28.676000000
H	31.990000000	32.796000000	29.129000000
H	31.518000000	26.632000000	28.177000000
H	33.848000000	30.498000000	29.620000000
H	31.352000000	24.118000000	26.344000000
H	34.023000000	33.000000000	31.460000000
H	31.196000000	27.014000000	25.200000000
H	34.175000000	30.129000000	32.595000000
H	33.849000000	27.803000000	24.144000000
H	31.514000000	29.329000000	33.658000000

Te_4C_4···Te_4C_4 chalcogen bonding motif
Energy (DFT-D3/BLYP/DZVP): -869100.670209632 kcal mol^{-1}
96

C	40.865000000	26.548000000	27.742000000
C	38.214000000	30.576000000	30.054000000
C	40.086000000	27.470000000	26.548000000
C	38.992000000	29.654000000	31.248000000
B	41.772000000	25.632000000	25.276000000
B	37.306000000	31.492000000	32.520000000
B	40.849000000	24.305000000	26.095000000
B	38.229000000	32.820000000	31.701000000
B	40.079000000	25.295000000	24.814000000
B	38.999000000	31.830000000	32.982000000
B	41.734000000	27.050000000	26.365000000
B	37.344000000	30.075000000	31.431000000
B	41.927000000	25.406000000	27.020000000
B	37.151000000	31.718000000	30.776000000
B	40.303000000	24.923000000	27.646000000
B	38.776000000	32.201000000	30.150000000
B	39.186000000	26.274000000	27.395000000
B	39.892000000	30.851000000	30.401000000
B	39.139000000	24.858000000	26.326000000
B	39.939000000	32.266000000	31.470000000
B	39.038000000	26.495000000	25.613000000
B	40.041000000	30.629000000	32.183000000
B	40.617000000	26.961000000	24.977000000
B	38.461000000	30.164000000	32.818000000

Te	41.531000000	27.338000000	29.631000000
Te	37.547000000	29.786000000	28.164000000
Te	39.292000000	27.509000000	31.088000000
Te	39.786000000	29.615000000	26.708000000
H	42.699000000	25.448000000	24.558000000
H	36.379000000	31.676000000	33.238000000
H	41.142000000	23.163000000	25.956000000
H	37.936000000	33.962000000	31.840000000
H	39.782000000	24.862000000	23.750000000
H	39.296000000	32.263000000	34.046000000
H	42.557000000	27.899000000	26.487000000
H	36.521000000	29.226000000	31.309000000
H	42.910000000	25.126000000	27.620000000
H	36.168000000	31.998000000	30.176000000
H	40.235000000	24.328000000	28.666000000
H	38.844000000	32.796000000	29.130000000
H	38.377000000	26.631000000	28.177000000
H	40.701000000	30.493000000	29.619000000
H	38.202000000	24.122000000	26.337000000
H	40.876000000	33.002000000	31.459000000
H	38.052000000	27.002000000	25.203000000
H	41.027000000	30.122000000	32.594000000
H	40.708000000	27.796000000	24.141000000
H	38.370000000	29.329000000	33.655000000
C	39.726000000	18.001000000	27.742000000
C	37.075000000	22.029000000	30.054000000
C	38.947000000	18.923000000	26.548000000
C	37.853000000	21.108000000	31.248000000
B	40.633000000	17.085000000	25.276000000
B	36.167000000	22.945000000	32.520000000
B	39.711000000	15.758000000	26.095000000
B	37.090000000	24.273000000	31.701000000
B	38.940000000	16.748000000	24.814000000
B	37.860000000	23.283000000	32.982000000
B	40.595000000	18.503000000	26.365000000
B	36.206000000	21.528000000	31.431000000
B	40.788000000	16.860000000	27.020000000
B	36.012000000	23.171000000	30.776000000
B	39.164000000	16.376000000	27.646000000
B	37.637000000	23.655000000	30.150000000
B	38.047000000	17.727000000	27.395000000
B	38.754000000	22.304000000	30.401000000
B	38.000000000	16.311000000	26.326000000
B	38.801000000	23.720000000	31.470000000
B	37.899000000	17.948000000	25.613000000
B	38.902000000	22.082000000	32.183000000
B	39.478000000	18.414000000	24.977000000
B	37.322000000	21.617000000	32.818000000
Te	40.392000000	18.791000000	29.631000000
Te	36.408000000	21.239000000	28.164000000
Te	38.153000000	18.962000000	31.088000000
Te	38.648000000	21.068000000	26.708000000

H	41.561000000	16.911000000	24.553000000
H	35.239000000	23.124000000	33.238000000
H	40.003000000	14.614000000	25.954000000
H	36.801000000	25.418000000	31.835000000
H	38.650000000	16.319000000	23.744000000
H	38.165000000	23.718000000	34.044000000
H	41.417000000	19.354000000	26.487000000
H	35.386000000	20.675000000	31.299000000
H	41.776000000	16.592000000	27.622000000
H	35.029000000	23.452000000	30.173000000
H	39.092000000	15.788000000	28.676000000
H	37.710000000	24.252000000	29.132000000
H	37.236000000	18.088000000	28.176000000
H	39.565000000	21.946000000	29.619000000
H	37.068000000	15.573000000	26.344000000
H	39.735000000	24.460000000	31.453000000
H	36.916000000	18.466000000	25.201000000
H	39.894000000	21.575000000	32.590000000
H	39.569000000	19.252000000	24.143000000
H	37.233000000	20.777000000	33.656000000

Se_4C_4 dimer stabilized via B-H···H-B contacts
Energy (DFT-D3/BLYP/DZVP): -12886866.640458 kcal mol^{-1}
96

C	10.191000000	26.751000000	30.346000000
C	10.792000000	22.473000000	32.466000000
C	11.820000000	26.495000000	30.713000000
C	9.163000000	22.729000000	32.099000000
Se	8.755000000	25.976000000	31.398000000
Se	12.228000000	23.247000000	31.414000000
Se	8.591000000	23.823000000	30.598000000
Se	12.392000000	25.400000000	32.214000000
B	11.639000000	27.011000000	28.010000000
B	9.344000000	22.212000000	34.802000000
B	12.772000000	26.638000000	29.309000000
B	8.211000000	22.586000000	33.503000000
B	12.586000000	28.318000000	28.782000000
B	8.397000000	20.905000000	34.029000000
B	11.053000000	27.968000000	31.201000000
B	9.930000000	21.256000000	31.611000000
B	9.921000000	28.371000000	29.878000000
B	11.062000000	20.852000000	32.934000000
B	9.990000000	27.064000000	28.685000000
B	10.993000000	22.160000000	34.127000000
B	11.170000000	25.846000000	29.264000000
B	9.813000000	23.378000000	33.548000000
B	12.706000000	27.942000000	30.518000000
B	8.277000000	21.282000000	32.294000000
B	11.522000000	29.147000000	29.965000000
B	9.461000000	20.076000000	32.847000000
B	10.873000000	28.588000000	28.395000000

B	10.109000000	20.636000000	34.417000000
H	11.635000000	30.292000000	30.260000000
H	9.348000000	18.931000000	32.552000000
H	10.506000000	29.334000000	27.549000000
H	10.476000000	19.890000000	35.263000000
H	11.831000000	26.631000000	26.901000000
H	9.152000000	22.592000000	35.911000000
H	13.726000000	25.944000000	29.232000000
H	7.257000000	23.280000000	33.581000000
H	13.480000000	28.867000000	28.225000000
H	7.503000000	20.356000000	34.586000000
H	10.818000000	28.117000000	32.350000000
H	10.165000000	21.107000000	30.462000000
H	8.879000000	28.856000000	30.167000000
H	12.104000000	20.368000000	32.645000000
H	13.612000000	28.134000000	31.257000000
H	7.371000000	21.090000000	31.555000000
H	8.999000000	26.668000000	28.175000000
H	11.984000000	22.556000000	34.637000000
H	11.010000000	24.684000000	29.167000000
H	9.973000000	24.540000000	33.646000000
C	16.330000000	37.205000000	30.346000000
C	16.931000000	32.927000000	32.466000000
C	17.959000000	36.949000000	30.713000000
C	15.302000000	33.183000000	32.099000000
Se	14.894000000	36.431000000	31.398000000
Se	18.368000000	33.701000000	31.414000000
Se	14.731000000	34.278000000	30.598000000
Se	18.531000000	35.854000000	32.214000000
B	17.778000000	37.466000000	28.010000000
B	15.483000000	32.667000000	34.802000000
B	18.911000000	37.092000000	29.309000000
B	14.350000000	33.040000000	33.503000000
B	18.726000000	38.772000000	28.782000000
B	14.536000000	31.360000000	34.029000000
B	17.192000000	38.422000000	31.201000000
B	16.069000000	31.710000000	31.611000000
B	16.060000000	38.826000000	29.878000000
B	17.201000000	31.306000000	32.934000000
B	16.129000000	37.518000000	28.685000000
B	17.132000000	32.614000000	34.127000000
B	17.309000000	36.300000000	29.264000000
B	15.952000000	33.832000000	33.548000000
B	18.845000000	38.396000000	30.518000000
B	14.416000000	31.736000000	32.294000000
B	17.661000000	39.601000000	29.965000000
B	15.601000000	30.531000000	32.847000000
B	17.013000000	39.042000000	28.395000000
B	16.249000000	31.090000000	34.417000000
H	17.772000000	40.747000000	30.263000000
H	15.488000000	29.386000000	32.550000000
H	16.641000000	39.794000000	27.552000000

H	16.619000000	30.334000000	35.259000000
H	17.969000000	37.079000000	26.901000000
H	15.290000000	33.050000000	35.912000000
H	19.866000000	36.390000000	29.239000000
H	13.397000000	33.740000000	33.582000000
H	19.618000000	39.330000000	28.227000000
H	13.642000000	30.808000000	34.585000000
H	16.954000000	38.569000000	32.354000000
H	16.304000000	31.562000000	30.461000000
H	15.018000000	39.310000000	30.176000000
H	18.242000000	30.822000000	32.640000000
H	19.755000000	38.579000000	31.259000000
H	13.512000000	31.543000000	31.552000000
H	15.137000000	37.118000000	28.174000000
H	18.124000000	33.015000000	34.641000000
H	17.147000000	35.138000000	29.167000000
H	16.108000000	34.996000000	33.645000000

Hypothetical **Se_4C_4** dimer stabilized via B-H···H-B contacts
Energy (DFT-D3/BLYP/DZVP): -12886866.175206 kcal mol^{-1}
96

C	10.099000000	26.594000000	30.346000000
C	10.700000000	22.316000000	32.466000000
C	11.728000000	26.338000000	30.713000000
C	9.071000000	22.572000000	32.099000000
Se	8.663000000	25.819000000	31.398000000
Se	12.136000000	23.090000000	31.414000000
Se	8.499000000	23.666000000	30.598000000
Se	12.300000000	25.243000000	32.214000000
B	11.547000000	26.854000000	28.010000000
B	9.252000000	22.055000000	34.802000000
B	12.680000000	26.481000000	29.309000000
B	8.119000000	22.429000000	33.503000000
B	12.494000000	28.161000000	28.782000000
B	8.305000000	20.748000000	34.029000000
B	10.961000000	27.811000000	31.201000000
B	9.838000000	21.099000000	31.611000000
B	9.829000000	28.214000000	29.878000000
B	10.970000000	20.695000000	32.934000000
B	9.898000000	26.907000000	28.685000000
B	10.901000000	22.003000000	34.127000000
B	11.078000000	25.689000000	29.264000000
B	9.721000000	23.221000000	33.548000000
B	12.614000000	27.785000000	30.518000000
B	8.185000000	21.125000000	32.294000000
B	11.430000000	28.990000000	29.965000000
B	9.369000000	19.919000000	32.847000000
B	10.781000000	28.431000000	28.395000000
B	10.017000000	20.479000000	34.417000000
H	11.543000000	30.135000000	30.260000000
H	9.256000000	18.774000000	32.552000000

H	10.414000000	29.177000000	27.549000000
H	10.384000000	19.733000000	35.263000000
H	11.739000000	26.474000000	26.901000000
H	9.060000000	22.435000000	35.911000000
H	13.634000000	25.787000000	29.232000000
H	7.165000000	23.123000000	33.581000000
H	13.388000000	28.710000000	28.225000000
H	7.411000000	20.199000000	34.586000000
H	10.726000000	27.960000000	32.350000000
H	10.073000000	20.950000000	30.462000000
H	8.787000000	28.699000000	30.167000000
H	12.012000000	20.211000000	32.645000000
H	13.520000000	27.977000000	31.257000000
H	7.279000000	20.933000000	31.555000000
H	8.907000000	26.511000000	28.175000000
H	11.892000000	22.399000000	34.637000000
H	10.918000000	24.527000000	29.167000000
H	9.881000000	24.383000000	33.646000000
C	16.422000000	37.362000000	30.346000000
C	17.023000000	33.084000000	32.466000000
C	18.051000000	37.106000000	30.713000000
C	15.394000000	33.340000000	32.099000000
Se	14.986000000	36.588000000	31.398000000
Se	18.460000000	33.858000000	31.414000000
Se	14.823000000	34.435000000	30.598000000
Se	18.623000000	36.011000000	32.214000000
B	17.870000000	37.623000000	28.010000000
B	15.575000000	32.824000000	34.802000000
B	19.003000000	37.249000000	29.309000000
B	14.442000000	33.197000000	33.503000000
B	18.818000000	38.929000000	28.782000000
B	14.628000000	31.517000000	34.029000000
B	17.284000000	38.579000000	31.201000000
B	16.161000000	31.867000000	31.611000000
B	16.152000000	38.983000000	29.878000000
B	17.293000000	31.463000000	32.934000000
B	16.221000000	37.675000000	28.685000000
B	17.224000000	32.771000000	34.127000000
B	17.401000000	36.457000000	29.264000000
B	16.044000000	33.989000000	33.548000000
B	18.937000000	38.553000000	30.518000000
B	14.508000000	31.893000000	32.294000000
B	17.753000000	39.758000000	29.965000000
B	15.693000000	30.688000000	32.847000000
B	17.105000000	39.199000000	28.395000000
B	16.341000000	31.247000000	34.417000000
H	17.864000000	40.904000000	30.263000000
H	15.580000000	29.543000000	32.550000000
H	16.733000000	39.951000000	27.552000000
H	16.711000000	30.491000000	35.259000000
H	18.061000000	37.236000000	26.901000000
H	15.382000000	33.207000000	35.912000000

H	19.958000000	36.547000000	29.239000000
H	13.489000000	33.897000000	33.582000000
H	19.710000000	39.487000000	28.227000000
H	13.734000000	30.965000000	34.585000000
H	17.046000000	38.726000000	32.354000000
H	16.396000000	31.719000000	30.461000000
H	15.110000000	39.467000000	30.176000000
H	18.334000000	30.979000000	32.640000000
H	19.847000000	38.736000000	31.259000000
H	13.604000000	31.700000000	31.552000000
H	15.229000000	37.275000000	28.174000000
H	18.216000000	33.172000000	34.641000000
H	17.239000000	35.295000000	29.167000000
H	16.200000000	35.153000000	33.645000000
